# Risk and Protective Factors for Tobacco Use Among 8th- and 10th-Grade African American Students in Virginia

**Published:** 2009-03-15

**Authors:** Rosalie Corona, Elizabeth Turf, Maya A. Corneille, Faye Z. Belgrave, Aashir Nasim

**Affiliations:** Department of Psychology, Virginia Commonwealth University; Virginia Commonwealth University, Richmond, Virginia; Virginia Commonwealth University, Richmond, Virginia; Virginia Commonwealth University, Richmond, Virginia; Virginia Commonwealth University, Richmond, Virginia

## Abstract

**Introduction:**

Few studies have simultaneously examined the influence of multiple domains of risk and protective factors for smoking among African Americans. This study identified individual-peer, family, school, and community risk and protective factors that predict early cigarette use among African American adolescents.

**Methods:**

Data from 1,056 African American 8th and 10th graders who completed the 2005 Community Youth Survey in Virginia were analyzed by using logistic regression.

**Results:**

The prevalence of smoking among the weighted sample population was 11.2%. In univariate analyses, the strongest predictors of smoking were low academic achievement, peer drug use, and early substance use (individual domain). In multivariate analyses, these factors and being in the 10th grade were significant predictors. The single protective factor in multivariate analyses was in the school domain (rewards for prosocial behavior in the school setting). When family and community variables were entered into a model in which individual-peer and school factors were controlled for, these variables were not significantly associated with smoking, and they failed to improve model fit.

**Conclusion:**

These findings suggest that tobacco prevention programs that aim to increase school connectedness while decreasing youth risk behaviors might be useful in preventing cigarette use among African American adolescents. Given the relative importance of peer drug use in predicting smoking among African American youth, more work is needed that explores the accuracy of youths' perceptions of their friends' cigarette use and how family factors may moderate this risk.

## Introduction

Tobacco use kills an estimated 438,000 people in the United States annually (1), and an estimated 8.6 million US adults had a smoking-related illness in 2000 (2). Of particular concern is smoking among youth, since many adult smokers began smoking as adolescents (3). Although the prevalence of cigarette use among youth has declined in recent years, results from the Monitoring the Future survey indicate that 22% and 35% of 8th- and 10th-grade students, respectively, reported in 2007 that they had ever smoked cigarettes (4). However, not all youth are equally susceptible to smoking (5). The prevalence of tobacco use differs among racial/ethnic groups; African American youth are less likely than other youth to use tobacco (4).

By late adolescence and early adulthood, tobacco use among African Americans increases (6,7). Because people who initiate tobacco use later in adolescence are less likely to experience smoking-related problems later in life (8), one would expect that African American smokers should experience fewer smoking-related health problems, since they begin smoking at older ages. However, this is not the case; African Americans are disproportionately affected by smoking-related illnesses and death (9), and once African Americans become daily smokers, they are less likely to quit than are other smokers (10,11). Therefore, preventing African American youth from starting smoking is a public health priority. Moreover, understanding the contextual factors associated with smoking in this group is also critical for evidence-based prevention programming.

Ecological models suggest that youth can be at risk for or protected from tobacco use because of individual, peer, family, school, and community factors. Many studies have explored such risk and protective factors among adolescents who report substance use, including tobacco use (6,12-14). For example, family factors (eg, parental nonsmoking, family monitoring, family bond) were associated with a lower risk of daily smoking among a diverse group of urban youth (15). Studies about the influence of peer substance use on youth cigarette use have produced mixed findings across different racial/ethnic groups. Specifically, peer tobacco use predicts smoking among white and Latino youth but not among African American youth (16). Finally, low school connectedness, academic difficulties, and neighborhood factors are associated with increased risk of smoking among diverse groups of adolescents (17-19).

Until recently, much of what was learned regarding the risk and protective factors associated with youth tobacco use came from studies of predominately white youth, and data are mixed regarding whether or not white and African American youth are vulnerable to the same risk factors (6,13). Moreover, the role of community factors is understudied relative to individual, peer, and family factors. Because of methodologic limitations (eg, small sample size, limitation in measurement), few studies have examined the influence of multiple domains simultaneously. We examined the relative contributions of individual, peer, family, school, and community risk and protective factors for smoking among African American youth, and we controlled for each domain simultaneously. Our findings may help in the development of culturally congruent, evidenced-based prevention programs for African American youth.

## Methods

### Study design and participants

We analyzed data from 1,056 8th- and 10th-grade African American youth who completed the 2005 Community Youth Survey in Virginia. The Community Youth Survey was based on the Communities That Care survey (20), which identifies risk and protective factors for alcohol, tobacco, and other drug use among youth. The survey collected basic demographic information and responses to compute 24 risk and 10 protective factors (20).

The Survey and Evaluation Research Laboratory of Virginia Commonwealth University collected data from Virginia public schools. Institutional review board approval was received from Virginia Commonwealth University, and parents and students were given the opportunity to opt out of the survey. Trained survey administrators went to the schools and worked with preselected classrooms to administer the surveys. They provided all students a paper survey and a pencil. Administrators read a prepared script aloud and told students that they could skip any questions that they did not want to answer. The anonymity of the survey was stressed, and students were instructed not to write their name anywhere on the survey. The survey consisted of 135 items that covered 4 domains: school, community, family, and individual-peer. Students responded with yes/no or Likert-type responses for the various items. To construct risk and protective factors, we combined multiple survey items into scales.

The Survey and Evaluation Research Laboratory collected data in the fall of 2005 (September through December). The Fairfax County Public School District also collected data the same year by using the same Community Youth Survey instrument. We merged and analyzed both sources of data. Initially, the state was stratified by health regions and then by a 2-stage (school-level and class-level) sampling process. Of the 60 districts identified, 31 high schools and 34 middle schools agreed to participate (51.7% and 56.7%, respectively). The resulting data were stratified by 5 health planning regions and clustered by 35 school districts in the state. Information regarding the study design and sampling method are available elsewhere (21).

We assessed survey responses for validity in 3 ways (20) and omitted any responses determined to be invalid. To allow for generalization, we weighted the data to full population numbers for schoolchildren in Virginia. Weighting adjusted for unequal chances of selection, differential response rates, and departures from key demographic variables. Full details regarding the cleaning, sampling, and weighting are available elsewhere (21). A total of 11,973 survey responses from 3 grade levels (8th, 10th, 12th) were obtained and determined to be valid. To explore patterns within a primarily younger group of African Americans, we only analyzed responses from 8th- and 10th-grade students who self-identified as black/African American.

### Measures

The risk and protective factors were calculated and organized into the 4 domains constructed by the developers of the Communities That Care survey (20): individual-peer, family, school, and community. We constructed the factors by combining 1 or more survey items. Most scales ranged from 0 to 4 or 1 to 5, and each 1-point increase indicated a 20% increase in risk or protection score. The single exception was the *early initiation of alcohol and marijuana* factor, which had a scale of 0 to 8, corresponding to the range of ages from 10 to 18 for initial exploration of drinking or smoking marijuana. More information regarding the Communities that Care Survey is available at http://ncadi.samhsa.gov/features/ctc/resources.aspx.

We made 2 changes to factors in the individual-peer domain because of the study's focus on cigarette use: 1) we removed the question, "How old were you when you first smoked a cigarette, even just a puff?" from the *early initiation of drugs* factor, and 2) we removed the question, "What are the chances you would be seen as cool if you smoked cigarettes?" from the *rewards for antisocial behavior* factor and included it in the *rewards for cigarette smoking* factor. We forced *rewards for cigarette smoking* into model 1 to assess possible confounding within the individual-peer and school domains.

Most of the factor scales showed good reliability; Cronbach α scores ranged from 0.71 to 0.84. Four scores were between 0.65 and 0.70: academic failure (0.66), rebelliousness (0.69), rewards for prosocial involvement (0.66), and belief in a moral order (0.68). Three had α between 0.50 and 0.60: early initiation of problem behavior (0.54), opportunities for prosocial involvement (0.58), and individual-peer social skills (0.57). Data needed to compute factors were missing from 3% to 16% of responses; the family domain had the highest proportion of missing data. Factors were treated as continuous variables in all statistical analyses.

Smoking was measured with the question, "How often have you smoked cigarettes during the last 30 days?" We dichotomized this variable such that any report of smoking in the past 30 days was recoded as smoking.

On the basis of prior research, we used sex, grade, and parental education as covariates. The education level for mothers and fathers was missing for 20% and 31% of the sample, respectively, and among those who did respond, 10% of both fathers and mothers had a postgraduate education. We categorized mother's education, the more complete of the 2 parental education measures, into 3 categories (high school diploma or less, some college or college degree, and postgraduate education) and used this variable in all models. Although use of this covariate resulted in a smaller sample size because of missing data, the fit of the models improved substantially.

### Data analysis

STATA version 10 (StataCorp LP, College Station, Texas) was used to analyze data, adjusting for the stratified and clustered sampling strategy and weighting and allowing for the use of the subpopulation estimation capability. The subpopulation estimation procedure allows analysis of a subpopulation of the data without affecting the variance estimation for the complete data file. Because data were found not to be missing at random (much higher frequency of missing responses for all variables related to the family), no imputation was done.

We used logistic regression to determine both univariate and multivariate associations with smoking. Variables with a univariate *P* value less than .20 were used as independent predictor variables to build the multivariate models. In model 1, risk and protective factors from the individual-peer and school domains with the largest odds ratios (ORs) in univariate analyses were used to build an additive model to identify which factors worked together to increase the odds for smoking. In model 2, we added family-level factors to model 1; in model 3, we added community-level factors to model 2. We also analyzed interaction terms between factors and either sex or grade; interaction terms did not significantly improve any models. We used log pseudolikelihood and goodness-of-fit measurements to assess model fit.

## Results

The final sample consisted of 1,056 African American students: 588 in the 8th grade and 468 in the 10th grade; 50.3% of 8th graders and 55.2% of 10th graders were girls. The mean age of respondents was 14.2 years (standard deviation, 1.2 years; range, 11-19 years). The prevalence of smoking among the weighted sample as a whole was 11.2% (Table 1). Prevalence of smoking did not differ by sex but nearly doubled from the 8th to the 10th grades. Prevalence of smoking decreased as mother's education increased; ratios of smoking among students whose mothers had a high school education or less were more than 5 times as high as those among students whose mothers had at least some postgraduate education.

In univariate analysis, academic failure was associated with the greatest risk for smoking; odds of smoking increased more than 4-fold with academic failure (Table 2). Friends' use of drugs conveyed the second greatest risk. Two family risk factors and 1 protective factor were significant in univariate analysis: parental attitudes favorable to antisocial behavior; parental attitudes favorable to alcohol, cigarette, and marijuana use; and family rewards for prosocial involvement. Only 1 of the community risk factors (perceived availability of drugs) was significantly associated with smoking.

In multivariate analysis, we retained only those variables that were significant at *P* < .20. Model 1 (Table 3) examines the combined effect on smoking of 11 risk and 4 protective factors from the individual-peer and school domains. Factors that predicated smoking included being in the 10th grade, doing poorly in school, having friends who use drugs, and using alcohol and marijuana at an early age. In terms of protective factors, increasing school rewards for prosocial involvement decreased the risk for smoking by 60%. Although the differences did not reach significance, increasing maternal education was protective against smoking. Interaction terms for sex or grade with risk and protective factors did not improve any of the models. The Hosmer and Lemeshow goodness-of-fit index was nonsignificant, which indicated good model fit.

Model 2 (Table 3) includes factors from the family domain. None of these factors significantly affected smoking after adjusting for individual-peer and school factors. Model 3 added both family- and community-level factors to model 1, although these did not affect the risk for smoking after adjusting for the individual-peer and school factors. Models 2 and 3 also had poorer fit and slightly lower pseudo *R* ^2^ compared with model 1.

## Discussion

In univariate and multivariate analyses, low academic achievement emerged as the strongest predictor of cigarette smoking in African American youth. Studies with youth from other racial/ethnic groups have also documented an association between academic difficulties and cigarette use (17,19), although the mechanism of this association is not clear (22). The stress and smoking literature suggests that smoking may be a means of coping with stress related to low academic achievement (23). Youth who experience difficulties in school may also be less engaged in or connected to their school than their peers, which may limit their exposure to school-level protective factors. We found that school rewards for prosocial involvement was the single protective factor associated with African American youth cigarette use. Together, these findings highlight the need to engage youth in prosocial behaviors in the school setting, which may improve academic achievement and prevent smoking.

Although some research suggests that peer modeling of substance abuse is more predictive of smoking among white adolescents than among African Americans (24), findings from our study highlight the association of peer drug use with smoking among African American youth. Adolescents who affiliate with drug-using peers may be pressured to smoke and use other illicit substances. This finding is consistent with the results of a recent study of African American adolescents that indicated that associating with risky peers (including peers who use drugs) is detrimental to academic engagement (25). Our peer drug-use measure, however, relies on youths' perceptions of their friends' drug use, which may be inaccurate. In a study of 2,277 African Americans at historically black colleges or universities, 90% overestimated their peers' use of cigarettes (26). These findings suggest that social marketing messages and prevention programs that accurately depict the prevalence of smoking among adolescents might be useful in smoking prevention interventions aimed at African American youth. More research is needed to examine whether young African Americans misperceive their peers' smoking and the effect of this on their own smoking habits. In addition, research is needed to identify the factors associated with misperceptions of peer smoking and to develop strategies to correct these misperceptions among African American youth.

Family- and community-level factors are also typically associated with smoking in African American youth (12,14,27). In this study, when family and community variables were entered into a model in which individual-peer and school factors were accounted for, these variables did not show a significant association with smoking, nor did they improve model fit. This finding is somewhat surprising given some research that suggests family is among the most influential factors that determines tobacco use among African American adolescents (27). However, our findings should not be taken to suggest that family and community factors are not related to smoking in African American youth. Instead, research must clarify how family and community factors interact with individual-peer and different aspects of academic factors. For example, one study showed that neighborhood disorganization predicts increases in urban African American adolescents' substance use, but this association was mediated in girls by attitudes and perceptions about drug use and harmfulness (18). In another study, family cohesion was predictive of academic interests and values but not academic effort after controlling for risky peer influence (25).

### Limitations

Though this study included simultaneous consideration of risk and protective factors in several domains, some limitations should be noted. First, 20% of students in this study did not report their mother's highest level of education and therefore were excluded from analyses. This exclusion may have resulted in a sample of youth from families with more education, particularly given that 10% of participants reported that their mother had some postgraduate education, and may limit generalizability to the general population of African American adolescents. Social desirability biases may also have affected participants' responses. Despite these limitations, this study is unique in that we examined the relative contributions of risk and protective factors for smoking among African American youth, while controlling for each domain simultaneously.

### Prevention implications

The identification of both academic and peer variables as risk and protective factors for cigarette smoking has implications for the development of effective prevention programs for African American youth. One method of promoting academic engagement among African American youth and decreasing their susceptibility to peer risk factors is to intervene directly; an alternative approach is to change youth attitudes and behaviors through their relationship with their parents. Programs that target African American youth smoking should promote positive identity development, self-efficacy, and prosocial peer relations. Prevention programs that involve parents should use culturally congruent methods to teach parents how to effectively communicate with their children about tobacco-related topics, promote positive and healthy relationships with their children, and increase monitoring of their children's activities, including knowing their children's friends. Culturally tailored prevention programs can increase African American youth (and parent) engagement and retention (6,28) and substance refusal skills (29). Culturally tailored programs reinforce cultural traditions, values, and histories; include lessons on cultural attributes such as ethnic identity and positive peer relationships; and make use of interdependent and relational methods. Programs that use relational and communal approaches to decrease youth substance use are likely to lead not only to new and positive peer relationships but also to improved academic achievement. Finally, although no differences in risk and protective factors by sex emerged in this study, other work has found that substance use among girls is associated with relationship issues (30). Therefore, developing culturally relevant, sex-based youth and family-based programs may be warranted (29,30).

## Figures and Tables

**Table 1:** Prevalence of Smoking by Demographic Characteristics Among African American 8th- and 10th-Grade Students (N = 1,056), Virginia, 2005^a^

**Characteristic**	Weighted % Who Smoked in Past 30 Days (95% CI)	Category %	*P* Value^b^
Total (N = 1,056)	11.2 (10.9-11.5)	100.0	NA
**Sex (15 unknown/missing)**			
Girls (n = 548)	10.6 (10.3-10.9)	50.5	Reference
Boys (n = 493)	11.8 (11.5-12.1)	49.5	.86
**Grade**			
8th (n = 588)	7.7 (7.5-8.0)	53.5	Reference
10th (n = 468)	15.1 (14.7-15.4)	46.5	.007
**Mother's education (192 unknown/missing)**			
High school graduate or less (n = 265)	18.5 (18.1-18.9)	40.3	Reference
Some college or college degree (n = 435)	7.1 (6.8-7.4)	47.8	.05
Postgraduate education (n = 164)	3.4 (3.2-3.5)	11.9	.03
**Father's education (315 unknown/missing)**			
High school graduate or less (n = 265)	7.9 (7.6-8.2)	50.3	Reference
Some college or college degree (n = 333)	7.9 (7.6-8.2)	39.5	.99
Postgraduate education (n = 143)	3.0 (2.8-3.2)	10.1	.35

Abbreviations: CI, confidence interval; NA, not applicable.

a Data collected from the 2005 Community Youth Survey in Virginia (25).

b Calculated by using Pearson χ^2^ test.

**Table 2:** Univariate Analysis of Risk and Protective Factors for Smoking Among 1,056 African American 8th- and 10th-Grade Students, Virginia, 2005

**Risk or Protective Factor**	n^a^	OR (95% CI)^b^	*P* Value
**Risk factors**			
Neighborhood attachment	1,008	1.34 (0.71-2.51)	.35
Community disorganization	988	1.34 (0.70-2.55)	.37
High community transition	970	1.19 (0.41-3.45)	.74
Community norms	1,000	1.97 (0.89-4.40)	.09
Perceived availability of drugs	1,011	1.79 (1.08-2.97)	.03
Perceived availability of hand guns	1,001	1.23 (0.77-1.98)	.37
Poor family management	923	1.51 (0.77-2.97)	.22
Family conflict	942	1.65 (0.96-2.85)	.07
Family history of antisocial behavior	934	1.51 (0.89-2.57)	.12
Favorable parental attitudes toward alcohol, cigarette, and marijuana use	948	2.06 (1.06-4.01)	.03
Favorable parental attitudes toward antisocial behavior	943	2.46 (1.32-4.57)	.006
Academic failure	1,000	4.26 (2.45-7.38)	<.001
Low commitment to school	1,040	2.40 (1.60-3.59)	<.001
Rebelliousness	1,050	2.21 (1.29-3.77)	.005
Early initiation of alcohol and marijuana^c^	1,022	1.57 (1.31-1.87)	<.001
Early initiation of problem behavior	1,034	1.43 (1.08-1.90)	.02
Favorable attitudes toward antisocial behavior	1,043	1.47 (0.65-3.33)	.34
Favorable attitudes toward drug use	1,042	2.24 (1.19-4.24)	.02
Perceived risks of alcohol, cigarette, and marijuana use	1,035	1.52 (0.80-2.88)	.19
Interaction with antisocial peers	1,032	2.25 (1.88-2.69)	<.001
Friends' use of drugs	1,032	2.84 (2.25-3.59)	<.001
Sensation seeking	1,032	1.65 (1.21-2.25)	.002
Rewards for smoking^d^	1,039	1.16 (0.87-1.54)	.31
Rewards for antisocial behavior^d^	1,038	1.21 (1.01-1.44)	.04
Gang involvement	1,038	1.06 (0.84-1.33)	.63
**Protective factors**			
Community opportunities for prosocial involvement	748	0.97 (0.53-1.77)	.91
Community rewards for prosocial involvement	993	0.83 (0.40-1.72)	.60
Family attachment	910	0.70 (0.37-1.30)	.25
Family opportunities for prosocial involvement	917	0.84 (0.48-1.47)	.53
Family rewards for prosocial involvement	912	0.58 (0.40-0.85)	.006
School opportunities for prosocial involvement	1,034	0.54 (0.30-0.97)	.04
School rewards for prosocial involvement	1,041	0.60 (0.30-1.20)	.15
Religiosity	963	0.77 (0.47-1.28)	.31
Social skills	1,042	0.34 (0.25-0.48)	<.001
Belief in a moral order	1,052	0.39 (0.21-0.72)	.004

Abbreviations: OR, odds ratio; CI, confidence interval.

a Because factor constructs relied on answers to multiple survey questions, a missing response on any component resulted in a missing value for that factor scale. Because of this variation, the reported n's are for students with complete data on the factor or factors reported.

b Simple logistic regression was used to determine OR. OR indicates the increase in odds associated with a 1-point increase in factor score.

c Factor modified to exclude cigarette smoking.

d *Rewards for antisocial behavior* split to create *rewards for smoking* as a separate factor.

**Table 3:** Multivariate Logistic Regression of Risk and Protective Factors for Smoking Among African American 8th- and 10th-Grade Students, Virginia, 2005

Variable	Model 1 (n = 784)^a^		Model 2 (n = 674)^a^		Model 3 (n = 663)^a^	

	OR (95% CI)^b^	*P* Value	OR (95% CI)^b^	*P* Value	OR (95% CI)^b^	*P* Value
**Grade**						
8th	1 [Reference]		1 [Reference]		1 [Reference]	
10th	3.39 (1.89-6.09)	<.001	4.04 (2.12-7.71)	<.001	5.22 (1.86-14.63)	.003
**Mother's education**						
High school graduate or less	1 [Reference]		1 [Reference]		1 [Reference]	
Some college or college degree	0.38 (0.12-1.17)	.09	0.31 (0.13-0.70)	.006	0.29 (0.13-0.69)	.006
Postgraduate education	0.24 (0.04-1.49)	.12	0.24 (0.05-1.18)	.08	0.23 (0.04-1.28)	.09
**Sex**						
Female	1 [Reference]		1 [Reference]		1 [Reference]	
Male	1.21 (0.35-4.24)	.75	1.29 (0.34-4.93)	.70	1.98 (0.44-8.86)	.36
**Risk and protective factors**						
Academic failure	3.34 (1.44-7.76)	.007	3.41 (1.12-10.37)	.03	2.73 (1.04-7.21)	.04
Friend's use of drugs	1.88 (1.21-2.92)	.007	1.45 (1.05-2.01)	.03	1.28 (0.93-1.76)	.12
Early initiation of alcohol and marijuana^c^	1.59 (1.24-2.04)	.001	1.59 (1.30-1.94)	<.001	1.52 (1.22-1.89)	<.001
Rewards for smoking^d^	1.38 (0.78-2.42)	.26	NI		NI	
Rewards for antisocial involvement^d^	0.52 (0.26-1.03)	.06	0.71 (0.43-1.16)	.16	0.81 (0.56-1.17)	.26
School rewards for prosocial involvement	0.42 (0.21-0.85)	.02	0.41 (0.25-0.69)	.001	0.37 (0.20-0.68)	.002
Parental attitudes favorable to antisocial behavior	NI		1.45 (0.57-3.68)	.42	1.23 (0.50-3.02)	.65
Parental attitudes favorable toward alcohol, cigarettes, and marijuana use	NI		1.07 (0.64-1.79)	.80	1.21 (0.75-1.94)	.42
Family conflict	NI		1.19 (0.46-3.10)	.71	1.25 (0.50-3.10)	.62
Family history of antisocial behavior	NI		1.07 (0.73-1.57)	.72	0.98 (0.64-1.50)	.92
Family rewards for prosocial involvement	NI		1.21 (0.46-3.18)	.70	1.07 (0.40-2.92)	.89
Perceived availability of drugs	NI		NI		1.36 (0.79-2.34)	.25
Community norms	NI		NI		0.70 (0.38-1.29)	.24
Pseudo *R* ^2^	.39		.38		.35	

Abbreviations: OR, odds ratio; CI, confidence interval; NI, not included in this model.

a Because factor constructs relied on answers to multiple survey questions, a missing response on any component resulted in a missing value for that factor scale. Information was particularly missing for items included in the family domain, which resulted in lower n's for models that included these variables. Because of this variation, the reported n's are for students with complete data on the factor or factors reported.

b For risk and protective factors, OR indicates the increase in odds associated with a 1-point increase in factor score.

c Factor modified to exclude cigarette smoking.

d *Rewards for antisocial behavior* split to create *rewards for smoking* as a separate factor.
